# High-resolution and highly accelerated MRI T2 mapping as a tool to characterise renal tumour subtypes and grades

**DOI:** 10.1186/s41747-024-00476-8

**Published:** 2024-07-10

**Authors:** Ines Horvat-Menih, Hao Li, Andrew N. Priest, Shaohang Li, Andrew B. Gill, Iosif A. Mendichovszky, Susan T. Francis, Anne Y. Warren, Brent O’Carrigan, Sarah J. Welsh, James O. Jones, Antony C. P. Riddick, James N. Armitage, Thomas J. Mitchell, Grant D. Stewart, Ferdia A. Gallagher

**Affiliations:** 1 Department of Radiology, University of Cambridge, Cambridge, CB2 0QQ UK; 2 The Institute of Science and Technology for Brain-Inspired Intelligence, Fudan University, Shanghai, China; 3 Department of Radiology, Addenbrooke’s Hospital, Cambridge University Hospitals NHS Foundation Trust, Cambridge, CB2 0QQ UK; 4 Sir Peter Mansfield Imaging Centre, University of Nottingham, Nottingham, UK; 5 Department of Pathology, Addenbrooke’s Hospital, Cambridge University Hospitals NHS Foundation Trust, Cambridge, CB2 0QQ UK; 6 Department of Oncology, Addenbrooke’s Hospital, Cambridge University Hospitals NHS Foundation Trust, Cambridge, CB2 0QQ UK; 7 Department of Urology, Addenbrooke’s Hospital, Cambridge University Hospitals NHS Foundation Trust, Cambridge, CB2 0QQ UK; 8 Department of Surgery, University of Cambridge, Cambridge, CB2 0QQ UK

**Keywords:** Carcinoma (renal cell), Kidney cortex, Kidney neoplasms, Magnetic resonance imaging, Oncocytoma (renal)

## Abstract

**Background:**

Clinical imaging tools to probe aggressiveness of renal masses are lacking, and T2-weighted imaging as an integral part of magnetic resonance imaging protocol only provides qualitative information. We developed high-resolution and accelerated T2 mapping methods based on echo merging and using *k*-t undersampling and reduced flip angles (TEMPURA) and tested their potential to quantify differences between renal tumour subtypes and grades.

**Methods:**

Twenty-four patients with treatment-naïve renal tumours were imaged: seven renal oncocytomas (RO); one eosinophilic/oncocytic renal cell carcinoma; two chromophobe RCCs (chRCC); three papillary RCCs (pRCC); and twelve clear cell RCCs (ccRCC). Median, kurtosis, and skewness of T2 were quantified in tumours and in the normal-adjacent kidney cortex and were compared across renal tumour subtypes and between ccRCC grades.

**Results:**

High-resolution TEMPURA depicted the tumour structure at improved resolution compared to conventional T2-weighted imaging. The lowest median T2 values were present in pRCC (high-resolution, 51 ms; accelerated, 45 ms), which was significantly lower than RO (high-resolution*;* accelerated, *p* = 0.012) and ccRCC (high-resolution, *p* = 0.019; accelerated, *p* = 0.008). ROs showed the lowest kurtosis (high-resolution, 3.4; accelerated, 4.0), suggestive of low intratumoural heterogeneity. Lower T2 values were observed in higher compared to lower grade ccRCCs (grades 2, 3 and 4 on high-resolution, 209 ms, 151 ms, and 106 ms; on accelerated, 172 ms, 160 ms, and 102 ms, respectively), with accelerated TEMPURA showing statistical significance in comparison (*p* = 0.037).

**Conclusions:**

Both high-resolution and accelerated TEMPURA showed marked potential to quantify differences across renal tumour subtypes and between ccRCC grades.

**Trial registration:**

ClinicalTrials.gov, NCT03741426. Registered on 13 November 2018.

**Relevance statement:**

The newly developed T_2_ mapping methods have improved resolution, shorter acquisition times, and promising quantifiable readouts to characterise incidental renal masses.

**Graphical Abstract:**

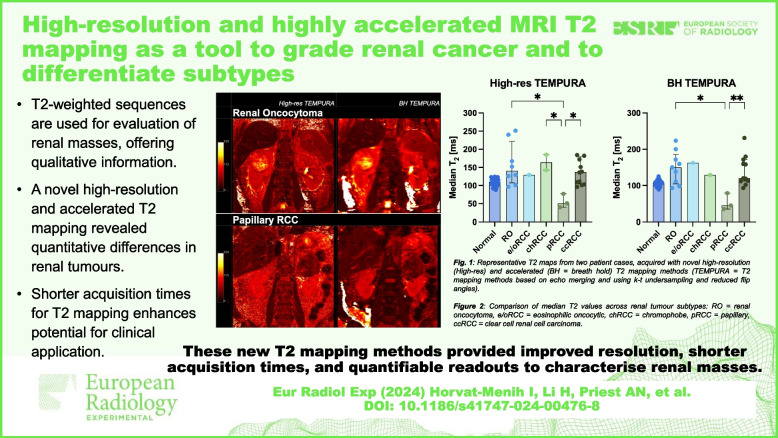

**Supplementary Information:**

The online version contains supplementary material available at 10.1186/s41747-024-00476-8.

## Key points

• T2-weighted sequences are used for evaluation of renal masses, offering qualitative information.

• This high-resolution and accelerated T2 mapping revealed quantitative differences in renal tumours.

• Shorter acquisition times for T2 mapping enhance potential for clinical application.

**Supplementary Information:**

The online version contains supplementary material available at 10.1186/s41747-024-00476-8.

## Background

Renal cell carcinoma (RCC) is the most lethal urological malignancy [1], with a reported 5-year cancer-specific survival of 0−32% in metastatic disease [2]. Early detection and accurate characterisation significantly impact the clinical management and improve survival [2, 3]. However, current diagnostic clinical imaging tools cannot accurately differentiate between grades and kidney tumour subtypes [4]. Renal mass biopsy (RMB) is invasive and nondiagnostic in up to 14% of cases [5]. A biopsy is also unrepresentative of the tumour heterogeneity which is characteristic of clear cell renal cell carcinoma (ccRCC) and can result in inaccurate grading or unnecessary surgery for benign lesions [5, 6]. Imaging overcomes some of the limitations of RMB including undersampling, but routine clinical imaging tools largely probe changes in size only [7, 8]. Therefore, novel imaging methods which noninvasively characterise the microstructure and biology of whole tumours have the potential to improve subtype differentiation and cancer grading.

In magnetic resonance imaging (MRI) T2 relaxation time denotes the time constant for decay of transverse magnetisation and is affected by molecular motion of the tissue of interest [9]. Large and bound molecules within solid tissues exhibit fast T2 relaxation, while small and rapidly moving molecules such as those present in extracellular free water, prolong T2 relaxation time [10]; T2-weighted images are an integral part of a clinical MRI protocol for evaluation of tumours [11]. In the kidney, T2 signal intensity of renal tumours has been compared to the renal cortex using a five-tier Likert scoring algorithm for the likelihood of a renal mass being a ccRCC, showing a good sensitivity (75%) and specificity (78%) when the score is 4 or 5 [12, 13]. However, T2-weighted imaging is only qualitative or semiquantitative, whereas T2 mapping provides robust quantification of T2 measurements which can be directly compared within and between patients [14]. In the kidney, T2 mapping is being tested as part of the clinical protocol to evaluate diffuse kidney disease [15], but has also shown promise in distinguishing high-grade from low-grade ccRCCs [16].

We have recently developed a novel approach for rapid and high-resolution T2 mapping to produce a pixel size of up to 0.75 × 0.75 mm^2^ in an acquisition time varying from 3 to 5 min to a single breath-hold (18 s). We have termed this method T2 mapping using the sequence Echo Merging Plus *k*-t Undersampling with Reduced refocusing flip Angles (TEMPURA) [17]. These improvements in spatial and temporal resolution offer the possibility of enhancing microstructural visualisation and this approach can be used as a routine tool in the future.

The purpose of this work was to probe the potential of novel T2 mapping methods to characterise renal tumour subtypes and grades. We report the quantification of T2 across a range of kidney tumour subtypes, while also confirming the previously reported differences in T2 across ccRCC grades [16]. In addition, we describe quantitative measures of intratumoural heterogeneity metrics, which have the potential to further improve the differentiation of kidney tumours.

## Methods

### Recruitment ethics and patients’ workflow

Patients with renal tumours presenting to Uro-oncology Clinic at Addenbrooke’s Hospital, Cambridge University Hospitals NHS Foundation Trust, Cambridge UK, between January 2022 and January 2023, were prospectively recruited and provided written informed consent for an ethically approved trial WIRE (WIndow-of-opportunity clinical trial platform for evaluation of novel treatment strategies in REnal cell cancer) (Research Ethics Committee: 19/LO/1461; ClinicalTrials.gov: NCT03741426) [18], and the IBM study (Investigation of differential biology of Benign and Malignant renal masses using advanced magnetic resonance imaging techniques) (Research Ethics Committee: 22/EE/0136).

Key inclusion criteria were ≥ 18 years, clinical suspicion of renal mass, Eastern Cooperative Oncology Group − ECOG performance status ≤ 1; and specifically for WIRE: biopsy-proven and surgically resectable ccRCC. Key exclusion criteria were unsuitability for MRI, significant comorbidities, pregnancy, immunosuppression, previous exposure to tyrosine kinase and poly ADP-ribose polymerase (PARP) inhibitors.

After the baseline scan, the patients recruited to the WIRE trial underwent RMB to determine the histology and WHO/ISUP (World Health Organisation/International Society of Urological Pathology) provisional grade [19]. In cases of ccRCC, the patient proceeded to the next stage of trial, undergoing neoadjuvant treatment. Depending on the clinical decision, all patients underwent either surgery or active surveillance.

### MRI acquisition: T2 mapping and T2-weighted sequence

Recruited patients underwent MRI using a 3-T scanner (Discovery MR750, GE Healthcare, WI, USA) and a 32-channel cardiac array coil, before any intervention (such as biopsy, treatment, or surgery). T2 maps were acquired by T2 mapping using Echo Merging Plus *k*-t Undersampling with Reduced flip Angles (TEMPURA), which is a multi-echo spin-echo-based method with a high acceleration factor (× 9) [17]. The scanned sequences employed were as follows: high-resolution (High-res) TEMPURA and breath-hold (BH) TEMPURA. acquisition parameters are shown in Table 1. Synthetic T2-weighted images were produced from the high-resolution T2 maps without additional acquisitions.

**Table 1:** TEMPURA acquisition parameters

Method	Acceleration	TE (min:ESP:max)	TR (ms)	Flip angle	Matrix	Bandwidth (Hz/pixel)	Acquisition time (min:s)
High-res TEMPURA	9 ×	7.8:7.8:234	1 breath (-5, 200)	175°–145°–110°–110°…	384 × 384	326	43 breaths (-3:45)
BH TEMPURA	9 ×	13:13:182	1,125	175°–145°–110°–110°…	128 × 128	488	0:18

Other parameters in common: field of view = 384 mm; 5 slices with thickness/gap of 4.5/1.0 mm. *TE* Echo time, *ESP* Echo spacing, *TR* Repetition time, *TEMPURA* T2 Mapping methods based on echo merging and using *k*-t undersampling and reduced flip angles

A separate T2-weighted CUBE sequence (three-dimensional fast spin-echo) was acquired for comparison, acquisition parameters were: field of view 360 mm; slice thickness 4.0 mm; echo time 100 ms, respiratory-triggering; flip angle 90°; matrix 256 × 224; and acquisition time -4 min.

### Processing and analysis of T_2_ maps

T2 maps were reconstructed using the *k*-t FOCUSS approach, with principal component analysis employed as the sparsifying transform [20, 21]. To account for stimulated echoes resulting from reduced refocusing flip angles, an extended phase graph model [22] was applied for T2 estimation. All processing was performed using MATLAB (MathWorks Inc., Natick, MA, USA).

Using OsiriX MD v.11 (Pixmeo SARL, Bernex, Switzerland), the regions of interest of tumours and adjacent normal-appearing renal cortex were manually drawn on processed T2 maps, with tumour regions of interest encompassing the largest diameter. Median T2, kurtosis, and skewness were extracted from each region of interest and compared across kidney tumour subtypes with normal-adjacent kidney serving as reference, and between ccRCC grades. The largest diameter was recorded for each analysed tumour and compared in the same fashion as T2 metrics.

### Statistical analysis

Statistical analysis of the quantified parameters was performed in GraphPad Prism v.10 (GraphPad Software, Boston, MA, USA). Shapiro–Wilk test was used to test for normality of data distribution, which prompted the choice of subsequent analyses. Agreement between the two novel T2 mapping methods, the High-res and the BH TEMPURA, was tested with Bland–Altman plots and Spearman correlations.

Comparisons across histological subtypes, across ccRCC grades, and between the largest tumour diameters were performed using the Kruskal–Wallis test with Dunn’s correction for multiple comparisons. Results were presented as median (range), and *p* < 0.05 was used as the cutoff for significance.

## Results

### Patient summary characteristics

A summary of the patient characteristics is shown in Table 2*.* In most patients, the renal mass was detected incidentally. Among the patients presenting with symptoms, three reported of unwanted weight loss in the previous six months, a further three noticed visible haematuria, two complained of flank pain and one patient complained of fatigue. On renal mass biopsy, an oncocytic neoplasm favouring a renal oncocytoma (RO) was discovered in seven patients, with two patients presenting with two lesions. Out of the seven patients, two opted for elective nephrectomy, which confirmed the RO as the final histology. An oncocytic renal neoplasm of low malignant potential, not otherwise specified as per WHO 2022 classification [23], was found on RMB in one patient: on the biopsy, this had features favouring the emerging entity of low-grade oncocytic tumour, but was subsequently reported as an eosinophilic/oncocytic RCC (e/oRCC) on final postsurgical histology, due to the presence of vascular invasion. Chromophobe RCC (chRCC) was confirmed in two patients, one of which harboured an additional ccRCC in the contralateral kidney which was subsequently surgically removed as a priority to chRCC. A further three patients harboured papillary RCC (pRCC), and altogether twelve (50.0% of the total) presented with ccRCC. Eight patients with ccRCC confirmed on RMB proceeded with neoadjuvant medication as part of the WIRE trial. Clinical decision for nephrectomy was made in 19 patients (79.2%). All but two patients with RO are under active surveillance. All but one of the twelve ccRCC patients underwent a RMB before surgery, and nephrectomy revealed a higher WHO/ISUP grade in ten patients (90.9%). This leaves only one patient with accurate grading at the time of RMB. Metastatic disease was discovered in four patients in the lungs, liver, brain, and hilar lymph nodes.

**Table 2:** Patient summary characteristics

Age at detection (median, range) (years)	63.5 (50−75)
Gender (female/male)	3/21
Presentation at detection (incidental/symptom-triggered investigation)	15/9
Histology of all tumours	
RO	7 (+ 2 due to bilateral)
LOT on RMB➔ e/oRCC on surgery	1
chRCC	2
pRCC	3
ccRCC	12
Clinical management	
Active surveillance	5
Nephrectomy	19
WHO/ISUP tumour grade on RMB and on surgery (ccRCC only, if RMB performed)	
Grade 1➔Grade 2	1
Grade 2➔Grade 2	1
Grade 2➔Grade 3	5
Grade 2➔Grade 4	2
Grade 3➔Grade 4	2
TNM stage at surgery (RCC only)	
(y)pT1a-b pNX cM0	4 (2 post-treatment)
(y)pT3a pNX cM0	7 (3 post-treatment)
(y)pT3a-b pNX-1 cM1	4 (2 post-treatment)
ypT4 pN0 cM0	1

*ccRCC* Clear cell RCC, *chRCC* Chromophobe RCC, *e/oRCC* Eosinophilic/oncocytic RCC, *LOT* Low-grade oncocytic tumour, pRCC Papillary RCC, *RMB* Renal mass biopsy, *RO* Renal oncocytoma, *RCC* Renal cell carcinoma

### Qualitative evaluation of T2 mapping results

**Fig. 1:**
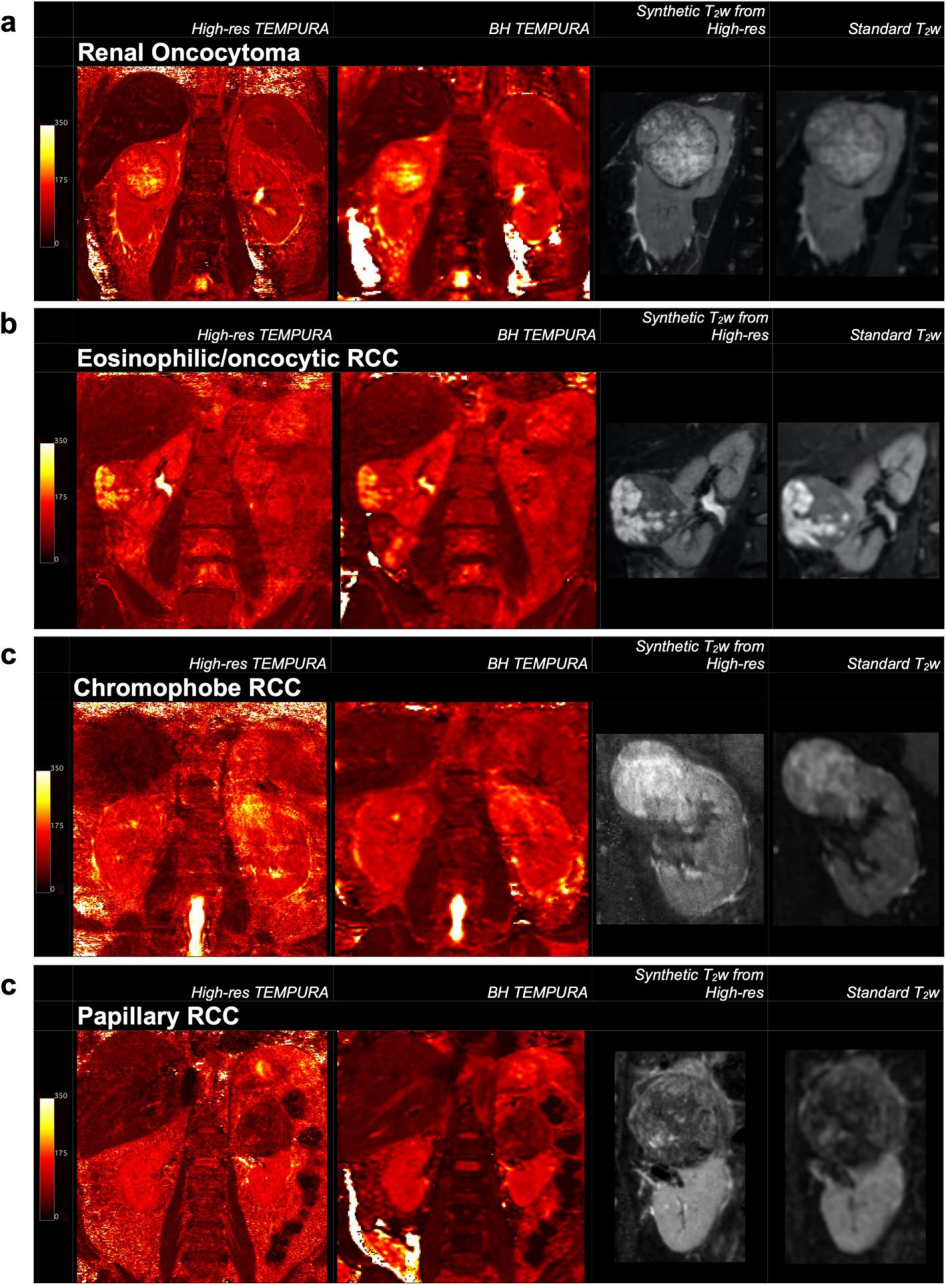
Representative T2 maps from the patient cohort. High-resolution TEMPURA, BH TEMPURA, synthetic T2-weighted from High-res TEMPURA and standard T2-weighted from: (**a**) renal oncocytoma; (**b**) eosinophilic/oncocytic RCC; (**c**) chromophobe RCC; and (**d**) papillary RCC. *TEMPURA* T2 mapping methods based on echo merging and using *k*-t undersampling and reduced flip angles; *RCC* Renal cell carcinoma

Representative T2 maps are shown in Fig. 1 for different kidney tumour subtypes. Synthetic T2-weighted images extracted from the high-resolution TEMPURA showed much greater microstructural detail and intratumoural heterogeneity compared to the standard T2-weighted acquisition. The e/oRCC demonstrated a clear demarcation between two distinct tumour regions on T2 mapping, one of which was haemorrhagic on pathology.

### Quantitative results extracted from T2 maps

**Fig. 2:**
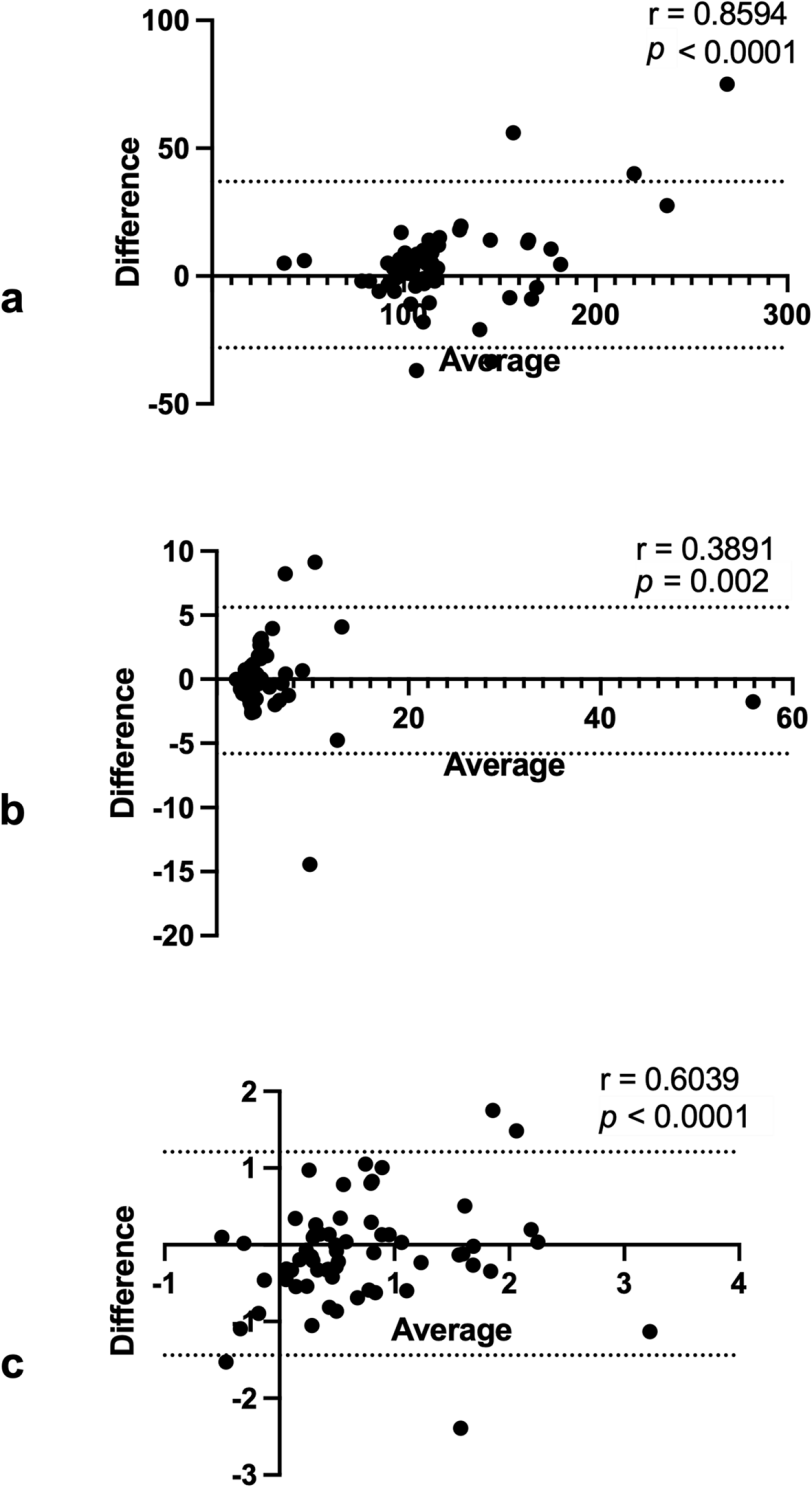
Bland–Altman plots with Spearman *r* and *p* values for testing agreement between the two T2 mapping methods, the High-res TEMPURA and BH TEMPURA, in kidney tumours and adjacent-normal kidneys, for the following metrics: (**a**) median, (**b**) kurtosis, and (**c**) skewness. *TEMPURA* T2 mapping methods based on echo merging and using *k*-t undersampling and reduced flip angles

High-res TEMPURA and BH TEMPURA T2 maps were in good agreement, as presented in Bland–Altman plots in Fig. 2, with Spearman correlations (*r*) of 0.86 (*p* < 0.0001), 0.39 (*p* < 0.01) and 0.60 (*p* < 0.001) for median, kurtosis and skewness, respectively.

**Fig. 3:**
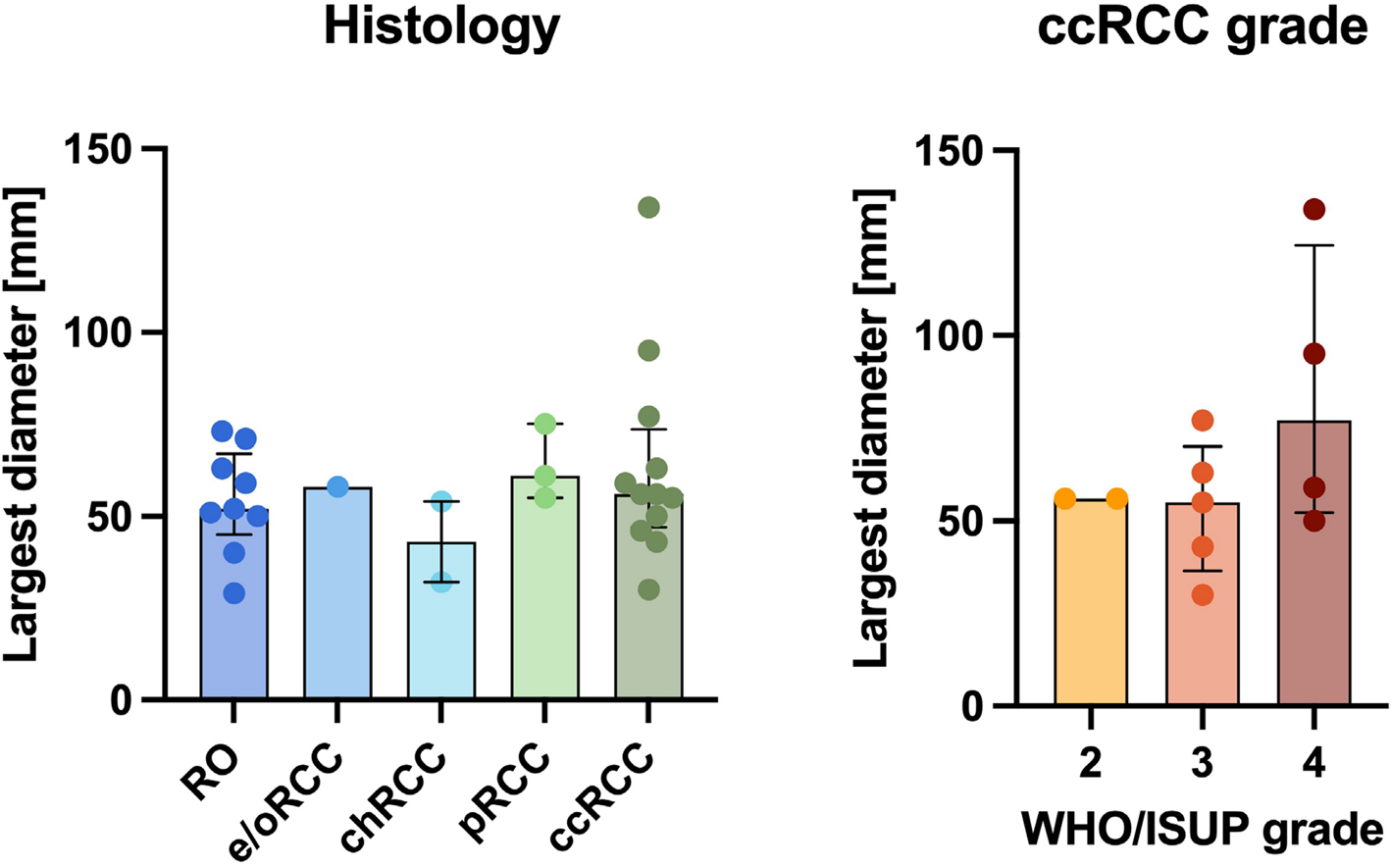
Largest diameters of tumours in the studied patient cohort, compared across histological subtypes, and between ccRCC WHO/ISUP grades. *ccRCC* Clear cell renal cell carcinoma, *chRCC* Chromophobe renal cell carcinoma, *e/oRCC* Eosinophilic/oncocytic renal cell carcinoma, *pRCC* Papillary renal cell carcinoma, *RO* Renal oncocytoma, *WHO/ISUP* World Health Organisation/International Society of Urological Pathology

**Fig. 4:**
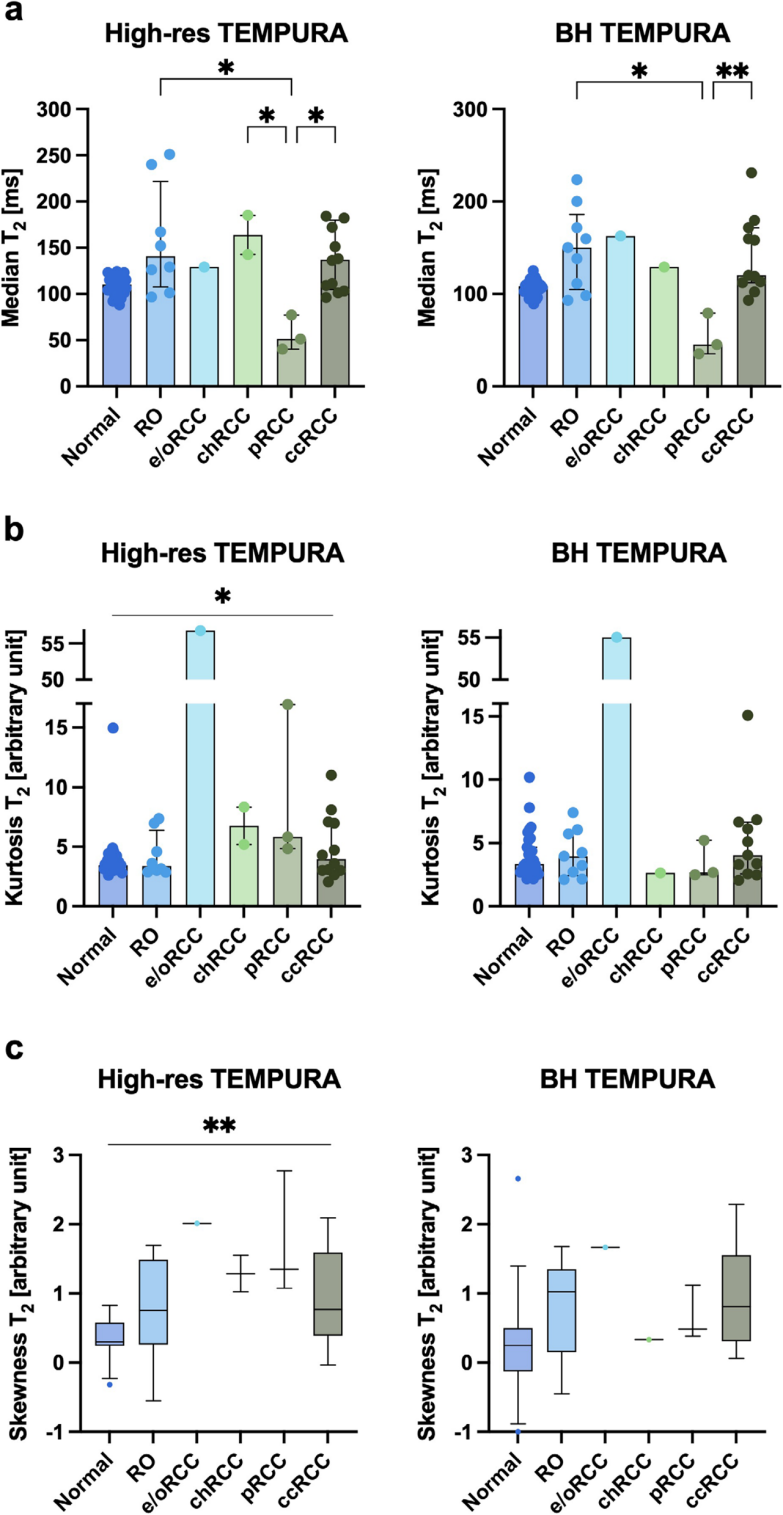
Bar plots and boxplots comparing High-res and BH TEMPURA-derived T2 values across kidney tumour subtypes. **a** Median T2 values (in ms). **b** Kurtosis (in arbitrary units). **c** Skewness (in arbitrary units). * *p* < 0.050; ** *p* < 0.010. *TEMPURA* T2 mapping methods based on echo merging and using *k*-t undersampling and reduced flip angles

**Fig. 5:**
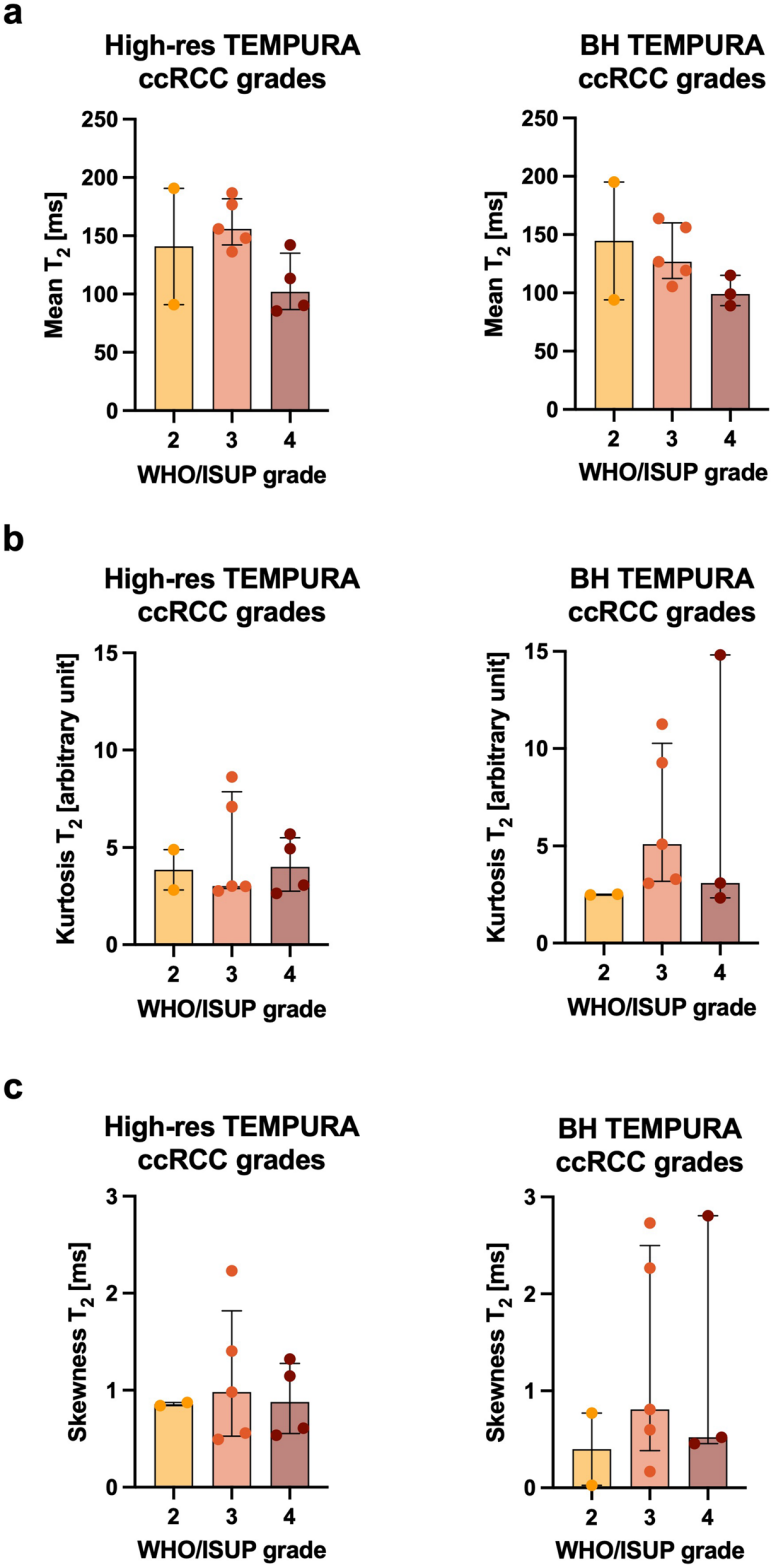
Bar plots comparing High-res and BH TEMPURA-derived T2 values across ccRCC WHO/ISUP grades. **a** Median T2 values (in ms). **a** Kurtosis (in arbitrary units). **c** Skewness (in arbitrary units). * *p* < 0.050; ** *p* < 0.010. *TEMPURA* T_2_ mapping methods based on echo merging and using *k*-t undersampling and reduced flip angles

Comparison of the largest tumour diameters across subtypes and between ccRCC WHO/ISUP grades are presented in Fig. 3, with numerical results recorded in Supplementary Table S1. Quantitative results for median, kurtosis and skewness of T2 values within the regions of interest are reported in Supplementary Table S2, and graphical representations are shown in Figs. 4 and 5.

#### Comparison across kidney tumour subtypes

Comparison of quantitative T2 metrics across kidney subtypes demonstrated that the lowest median T2 was in pRCC (high-res 51 ms; BH 45 ms), which was significantly lower than RO (*p* = 0.012), chRCC (*p* = 0.021), and ccRCC (*p* = 0.019) lesions on High-res TEMPURA, and RO (*p* = 0.012) and ccRCC (*p* = 0.008) on BH TEMPURA acquisitions (Fig. 4a).

The other RCCs and ROs presented with a high median T2 and a high interpatient variation as measured by the range relative to normal, however no comparison was significant. Kurtosis and skewness of the T2 distributions were used as measures of heterogeneity and were highest in the e/oRCC case, which corresponded to the high level of visual heterogeneity identified on qualitative evaluation (Fig. 1). As expected, this was preferentially detected by High-res TEMPURA rather than BH TEMPURA, due to the larger number of voxels (Fig. 4b,c). Interestingly, ROs exhibited the lowest kurtosis (High-res: 3.4; BH: 4.0) which was comparable to the normal cortex (High-res: 3.4; BH: 3.3), suggestive of low intratumoural heterogeneity. This was in contrast to the high interpatient variation as determined by the median T2 (141 ms), with a relatively large range (97–251 ms), as presented in Fig. 4a.

The comparison of the largest tumour diameters across subtypes revealed a relatively homogeneous patient cohort, within the range typical for renal masses (median 56 mm, range 29–134 mm), as presented in Fig. 3. The smallest tumour was a RO and the largest was a ccRCC; however, no comparison was statistically significant (*p* = 0.566).

#### Comparison between ccRCC WHO/ISUP grades

The final postsurgical grading was used for the comparison of WHO/ISUP grades within the ccRCC group and the pre-treatment baseline scans. This revealed a decreasing T2 median from WHO/ISUP Grade 2 to 4 on both TEMPURA sequences (grades 2, 3, and 4, on High-res, 209 ms, 151 ms, and 106 ms; on BH, 172 ms, 160 ms, and 102 ms, respectively), with the BH TEMPURA showing statistical significance in comparison (*p* = 0.037) (Fig. 5a). A trend of increasing kurtosis and skewness was observed from lower to higher grades, as expected and shown in Fig. 5b, c. In the same comparison, the largest diameter appeared slightly larger in higher grades, but this was not statistically significant (Fig. 3).

## Discussion

In this study, we describe and test novel T2 mapping methods for imaging renal tumours. The high-resolution method improved the depiction of microstructure and intratumoural heterogeneity, which could offer new insights into tumour biology compared to conventional T2-weighted methods. The BH TEMPURA approach reduced the acquisition time to a single breath hold, and therefore this could be easily integrated into the clinical pathway. Additionally, we characterised whole-tumour heterogeneity, which could help to overcome the potential sampling error of a single biopsy, as demonstrated by the cases that were undergraded on RMB compared to surgical pathology. This method could be used in the future to guide a biopsy more accurately to areas of higher grade based on the lowest T2 value in the tumour. Furthermore, both TEMPURA sequences provided comparable quantitative readouts which could be used in the future to assess kidney tumour subtype differentiation and grade prediction.

Here we report quantification of T2 metrics across a range of different kidney tumour subtypes. The lowest median T2 was present in pRCC, corresponding to isointense or hypointense T2 signal intensity compared to the renal cortex on the clear cell likelihood score (ccLS) v2.0 [12]. The median T2 was relatively high in all other tumours, also consistent with the above-mentioned scoring, which describes these tumours as iso- to hyperintense compared to the renal cortex [12]. In addition, ROs exhibited high variation in median T2 between patients, but low intratumoural heterogeneity within lesions. Further studies are required to confirm these findings in larger cohorts, and correlate with longitudinal potential for progression [24]. Low median T2 was measured in high-grade ccRCCs compared to lower-grade tumours, which is in agreement with a previous report [16]. A trend for increasing intratumoural heterogeneity measures was detected from lower to higher grades as expected, although this was not statistically significant and again could be explored in larger cohorts. This approach has the potential to predict whole-tumour aggressiveness non-invasively and prioritise patients for treatment.

To further corroborate the clinical relevance of the novel T2 mapping methods presented here, several limitations of this study will need to be addressed. The studied patient cohort was relatively small, and the performance of the newly developed T2 mapping methods was not compared to other quantitative MRI methods that have been more extensively studied for their potential in renal tumour subtyping, such as dynamic contrast-enhanced MRI [25], diffusion-weighted imaging [26] and blood oxygenation level-dependent MRI [27]. Ideally, therefore, future studies will require larger patient cohorts as well as a study design including a comparison of diagnostic performance between different quantitative MRI techniques. Finally, a limitation from the analysis point of view was the quantification of the T2 values on a single coronal slice, which could have obscured variation of the imaging signal within the tumour volume.

In conclusion, we have shown that novel T2 mapping methods could have potential in different clinical scenarios for the management of kidney tumours, including subtype differentiation and grade prediction. Importantly, imaging has the potential to provide non-invasive quantitative whole-tumour intratumoural heterogeneity metrics. These may aid treatment stratification by combining the results from a single biopsy. Future studies should evaluate a larger number of patients to validate these findings and test the value of the novel T2 mapping methods in a routine clinical scenario.

### Supplementary Information

Supplementary Material 1: Supplementary Table 1. Largest diameters in millimeters derived from respective ROIs of High-res and BH TEMPURA acquisitions, and statistical results for comparisons across histological subtypes and ccRCC grades. Results are is shown as median (range). Supplementary Table 2. Quantitative T2 metrics derived from respective ROIs of High-res and BH TEMPURA acquisitions, and statistical results for comparisons across histological subtypes, and ccRCC grades.

## Data Availability

The datasets analysed during the current study are not publicly available due to the clinical trial still ongoing, but are available from the corresponding author on reasonable request.

## Abbreviations

BH: Breath-hold
ccRCC: Clear cell renal cell carcinoma
chRCC: Chromophobe renal cell carcinoma
e/oRCC: Eosinophilic/oncocytic renal cell carcinoma
High-res: High-resolution
pRCC: Papillary renal cell carcinoma
RCC: Renal cell carcinoma
RMB: Renal mass biopsy
RO: Renal oncocytoma
TEMPURA: T2-mapping using Echo Merging Plus k-t Undersampling with Reduced refocusing flip Angles
WHO/ISUP: World Health Organisation/International Society of Urological Pathology
WIRE: WIndow-of-opportunity clinical trial platform for evaluation of novel treatment strategies in REnal cell cancer
