# Supplementary material for: High-resolution and highly accelerated MRI T2 mapping as a tool to characterise renal tumour subtypes and grades
Source: Eur Radiol Exp. 2024 Jul 10;8:76. doi: 10.1186/s41747-024-00476-8 (PMC11233479; doi:10.1186/s41747-024-00476-8)
Supplement: Supplementary file 1 — Supplementary Material 1: Supplementary Table 1. Largest diameters in millimeters derived from respective ROIs of High-res and BH TEMPURA acquisitions, and statistical results for comparisons across histological subtypes and ccRCC grades. Results are is shown as median (range). Supplementary Table 2. Quantitative T2 metrics derived from respective ROIs of High-res and BH TEMPURA acquisitions, and statistical results for comparisons across histological subtypes, and ccRCC grades. [file 41747_2024_476_MOESM1_ESM.pdf]

High-resolution and highly accelerated MRI T2 mapping as a tool to characterise renal tumour subtypes and grades

ELECTRONIC SUPPLEMENTARY MATERIAL

Supplementary Table 1. Largest diameters in millimeters derived from respective ROIs of High-res and BH TEMPURA acquisitions, and statistical results for comparisons across histological subtypes and ccRCC grades. Results are is shown as median (range).

	RO	e/oRCC	chRCC	pRCC	ccRCC	Kruskal-Wallis *P*
**Largest diameter** [mm]	52 (29-73)	58 (one case)	43 (32-54)	61 (55-75)	56 (30-134)	0.566

	Grade 2	Grade 3	Grade 4	Kruskal-Wallis *P*
**Largest diameter** [mm]	56 (56-56)	55 (30-77)	77 (50-134)	0.442

Supplementary Table 2. Quantitative T2 metrics derived from respective ROIs of High-res and BH TEMPURA acquisitions, and statistical results for comparisons across histological subtypes, and ccRCC grades.

	Normal-adjacent	RO	e/oRCC	chRCC	pRCC	ccRCC	Kruskal-Wallis *P*
**High-res TEM-PURA**							
Median	110 (88-124)	141 (97-251)	129	164	51 (40-77)	137 (96-306)	0.001
Kurtosis	3.4 (2.6-1.5)	3.4 (2.9-7.4)	57	6.8 (5.2-8.3)	5.8 (4.8-17)	3.9 (2.0-11)	0.037
Skewness	0.3 (-0.3-3.8)	0.8 (-0.6-1.7)	2.0	1.3 (1.0-1.5)	1.3 (1.1-2.8)	0.8 (-0.0-2.1)	0.006
**BH TEMPURA**							
Median	108 (89-125)	150 (93-224)	163	129	45 (35-79)	120 (93-231)	0.001
Kurtosis	3.3 (2.2-10.2)	4.0 (2.1-7.4)	55.0	2.6	2.7 (2.5-5.2)	4.1 (2.1-15.1)	0.379
Skewness	0.3 (-1.2-2.7)	1.0 (-0.5-1.7)	1.7	0.3	0.5 (0.4-1.1)	0.8 (0.1-2.3)	0.051

	Grade 2	Grade 3	Grade 4	Kruskal-Wallis *P*
**High-res TEM-PURA**				
Median	209 (111-306)	151 (101-184)	106 (96-136)	0.170
Kurtosis	2.8 (2.0-3.6)	4.3 (3.0-8.1)	5.9 (2.6-11)	0.398
Skewness	0.06 (-0.03-0.16)	1.36 (0.35-1.82)	1.29 (0.61-2.09)	0.071
**BH TEMPURA**				
Median	172 (113-231)	160 (119-180)	102 (93-112)	0.037
Kurtosis	2.7 (2.0-3.4)	5.1 (3.3-6.8)	6.7 (2.6-15)	0.321
Skewness	0.6 (0.3-1.0)	0.8 (0.2-1.9)	1.5 (0.1-2.3)	0.886

Units of median values are milliseconds, while kurtosis and skewness have arbitrary units. All results are presented as median (range), with exception of *P* values in the last column.
